# The Endoscopic Versus Open Approach for Anterior Skull Base Tumors: A Systematic Review of Comparative Outcomes and a Framework for Surgical Selection

**DOI:** 10.1155/nri/7730393

**Published:** 2025-11-14

**Authors:** Ubaid Ullah Mian, Alishba Hameed, Touba Azeem, Sajjad Ullah, Muhammad Idris Khan, Hammad Iftikhar, Umar Farooq, Meer Wais, Jibran Ikram

**Affiliations:** ^1^Khyber Medical College, Peshawar, Khyber Pakhtunkhwa, Pakistan; ^2^Department of Neurosurgery, Khyber Teaching Hospital, Peshawar, Khyber Pakhtunkhwa, Pakistan; ^3^Department of ENT, Saidu Group of Teaching Hospital, Swat, Khyber Pakhtunkhwa, Pakistan; ^4^Department of Emergency Medicine, Royal Blackburn Teaching Hospital, East Lancashire, Haslingden Rd, Blackburn BB2 3HH, UK; ^5^Cardiovascular Medicine Department, Heart, Vascular and Thoracic Institute, Cleveland Clinic, Cleveland, Ohio, USA

**Keywords:** anterior skull base tumors, decision-making framework, endoscopic approach, open surgery, surgical complications, systematic review, tumor classification

## Abstract

**Background:**

Anterior skull base tumors (ASBTs) pose significant surgical challenges due to their proximity to critical neurovascular structures. Surgical management has evolved with the adoption of both endoscopic and open approaches. This systematic review synthesizes evidence comparing these approaches in terms of complications, outcomes, and indications.

**Methods:**

We conducted a systematic review following the PRISMA guidelines, analyzing studies published between 1981 and 2022. A total of 1200 articles were initially identified from databases including PubMed, MEDLINE, JSTOR, and ScienceDirect, with 60 relevant references ultimately included. Data extraction focused on surgical approaches, tumor types, prevalence, and complications.

**Results:**

ASBTs exhibit varying prevalence and associated complications depending on their type. Meningiomas account for nearly one-third of all cases, with an annual incidence of 2 per 100,000 individuals and recurrence rates ranging from 5% for Grade I to 50%–80% for Grade III. Common complications include anosmia (10%–20%), cerebrospinal fluid (CSF) leakage (10%), visual abnormalities, and bleeding (5%–10%). Pituitary adenomas are predominantly secretory, with microadenomas comprising 97% and macroadenomas 70%. They frequently cause damage to the internal carotid artery, optic nerve, and result in CSF leakage. Craniopharyngiomas are reported at 0.1 cases per 100,000 annually, with over 80% situated in the suprasellar region. Cavernous sinus tumors represent less than 3% of all meningiomas, while glomus tumors, more prevalent in females (6:1 ratio), present 1–3 instances per million individuals and can lead to facial paralysis, auditory impairment, and cranial nerve palsies. Chordomas and chondrosarcomas, occurring at 0.08 cases per 100,000, are more common in Caucasian men. Esthesioneuroblastomas constitute 2%-3% of intranasal neoplasms, often resulting in CSF leakage and infection. Craniofacial malignancies predominantly originate from the maxillary (60%–70%) and ethmoid sinuses (10%–15%), while skull base metastasis appears in approximately 4% of cancer patients, typically from breast, lung, renal, and prostate cancers. Surgical approaches also come with distinct complications. The endoscopic endonasal approach (EEA) has a bacterial meningitis rate of 0%–0.69%, with venous thromboembolism (VTE) being rare but more likely in older patients or those with coagulation issues. Cerebral infarction may occur due to vasospasm, subarachnoid hemorrhage, vascular damage, or electrolyte imbalances, while the risk of pneumocephalus is minimized through careful lumbar drain management and sinus precautions. Open surgical approaches commonly result in CSF leaks, meningitis, vascular injury, and visual disturbances.

**Conclusion:**

This systematic review synthesizes evidence from 60 studies to propose a decision-making framework. We conclude that the EEA is associated with superior quality of life and reduced morbidity for midline tumors (e.g., tuberculum sellae meningiomas and pituitary adenomas), offering comparable gross total resection rates. In contrast, open approaches remain paramount for tumors with significant lateral extension, massive size, or complex vascular involvement, where maximal exposure facilitates radical resection despite higher associated morbidity. This analysis provides a nuanced evidence base to guide individualized surgical strategy.

## 1. Introduction

Anterior skull base tumors (ASBTs) include a wide range of neoplasms [1] that originate anatomically in the area between the skull base and facial structures. These tumors present considerable surgical challenges owing to their proximity to eloquent neurovascular structures, such as the brain, optic nerves and chiasm, and the internal carotid arteries (ICAs). With time, significant breakthroughs in surgical procedures have brought about a revolutionary transformation in the treatment of malignant tumors. There are two main techniques often used for the resection of tumors, namely, endoscopic [2] and open procedures [3]. Each of these approaches has unique benefits and concerns.

Endoscopic procedures include the use of minimally invasive methodologies, which entail employing tiny incisions and specialized devices to access and extract the tumor. These methodologies frequently use endoscopes, providing exceptional viewing and magnification capabilities inside the surgical domain. Compared to conventional open techniques, they provide advantages such as decreased morbidity, shorter hospitalization durations, and expedited recovery periods. The use of endoscopic procedures has become more prevalent in the treatment of cancers situated inside the nasal cavity, paranasal sinuses, and suprasellar area [2].

Open surgical approaches often necessitate larger incisions and may involve the removal of bone to achieve adequate exposure of the tumor. These techniques allow for direct visualization and access to the tumor, facilitating complete resection while minimizing the risk of injury to adjacent critical structures. Open procedures are particularly indicated for larger tumors involving complex anatomical regions such as the anterior cranial fossa, cribriform plate, and ethmoid sinuses, where extensive exposure is required for safe and effective tumor removal [3].

Tumors of the anterior skull base encompass a wide variety of histological types, including meningiomas, esthesioneuroblastomas, chordomas, chondrosarcomas, and squamous cell carcinomas. Among these, meningiomas are the most frequently encountered, accounting for approximately 30% of all cases in this region. These tumors often present unique challenges due to their proximity to critical neurovascular structures, necessitating precise surgical approaches for effective management [4]. Meningiomas often originate from the dura mater and may exhibit either benign or malignant characteristics. Esthesioneuroblastomas are an infrequent kind of neoplasms that originate from the olfactory epithelium. These tumors represent a lesser but noteworthy proportion of cancers located in the anterior skull base.

The incidence of these cancers exhibits variability based on the particular histological subtype and geographical region. As an example, it is seen that meningiomas have a higher prevalence among females. Still, esthesioneuroblastomas display a bimodal age distribution characterized by two distinct peaks occurring throughout infancy and middle life. The prevalence of ASBTs is generally modest, with estimated rates ranging from 1 to 5 instances per 100,000 individuals [1].

The surgical resection of ASBTs is inherently associated with significant risks and potential complications. These include injury to critical neurovascular structures such as the optic nerves, carotid arteries, and frontal lobes, which can lead to visual impairment, cerebrospinal fluid (CSF) leakage, hemorrhage, infection, and neurological deficits [4]. The severity of these complications is influenced by factors such as tumor size, location, invasiveness, and the specific surgical approach employed. While advancements in preoperative imaging, intraoperative monitoring, and surgical technique have been crucial in reducing morbidity and improving outcomes, a definitive consensus on the optimal surgical strategy remains elusive.

Despite these advancements, the choice between endoscopic and open approaches for ASBTs is often debated, centering on the balance between achieving maximal safe resection and minimizing postoperative morbidity. A clear, evidence-based synthesis that compares the outcomes, risks, and specific indications for these approaches across the spectrum of ASBTs is lacking. Therefore, this systematic review aims to critically synthesize the existing literature to address a central clinical question: For which ASBTs and under what conditions does the endoscopic approach provide a superior risk-benefit profile, and when does the traditional open approach remain the gold standard? To answer this, we first posed the following research question: *‘How do endoscopic and open surgical approaches compare in terms of tumor classification, prevalence, and associated surgical complications?'* The ultimate goal is to distill the current evidence into a practical framework to aid in preoperative surgical planning and decision-making.

## 2. Materials and Methods

### 2.1. Research Question

How do endoscopic and open surgical approaches compare in terms of tumor classification, prevalence, and associated surgical complications in the management of ASBTs?

### 2.2. Search Strategy

We conducted an extensive search across a multitude of scholarly databases, including PubMed MEDLINE, JSTOR, ScienceDirect, the Cochrane Library, and Google Scholar, in order to comprehensively review articles pertaining to the chosen subject matter and its associated MeSH terms, such as “endoscopic and open approach,” “ASBTs,” and “surgical complications.” Search strategies of the databases are mentioned in Supporting File 1.

### 2.3. Study Selection and Eligibility Criteria

Our initial database search yielded 1200 records from 1981 through 2022. After compiling the search results, 720 duplicate entries were removed, resulting in 480 unique articles for screening. The titles and abstracts of these 480 articles were screened for relevance, which led to the exclusion of 422 records. A total of 60 studies were included in the final qualitative synthesis. The eligibility of each potentially relevant study was then independently assessed by two reviewers based on the predefined inclusion criteria. Discrepancies between reviewers were resolved through discussion and consensus with a third author. Eligible studies for this systematic review met the following criteria: (1) Study type: Review articles, case studies, retrospective studies, and clinical trials; studies published between 1981 and 2022; and articles following the Preferred Reporting Items for Systematic Reviews and Meta-Analyses (PRISMA) guidelines or similar methodological frameworks. (2) Population: Patients diagnosed with ASBTs, including meningiomas, esthesioneuroblastomas, pituitary adenomas, craniopharyngiomas, and chordomas. (3) Interventions: Studies comparing endoscopic and open surgical approaches and studies addressing surgical management, including tumor resection and complications. (4) Outcomes: Tumor prevalence and classification. Postsurgical complications, including CSF leaks, carotid injuries, anosmia, meningitis, and infection and clinical presentations, diagnostic findings, and recurrence rates. (5) Language and accessibility: Articles published in English and full-text articles accessible through databases such as PubMed, JSTOR, and ScienceDirect. (6) Date range: Studies published between 1981 and 2022. Exclusion criteria were as follows: (1) Nonsystematic reviews, editorials, commentaries, and opinion pieces and studies not adhering to robust methodological standards (e.g., incomplete or poorly documented data). (2) Studies involving tumors outside the boundary of the anterior skull base. (3) Studies exclusively focusing on radiation therapy, chemotherapy, or other nonsurgical treatments. (4) Articles lacking data on complications, tumor classification, or prevalence rates and studies with insufficient statistical analysis or unclear conclusions. (5) Non-English articles or those without available translations. Abstract-only articles or those requiring subscription access if full-text is unavailable. (6) Studies published before 1981 or after 2022.

**Figure 1:**
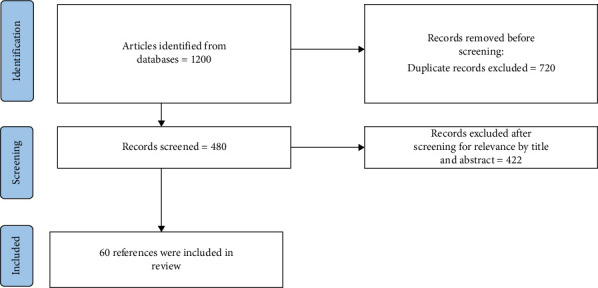
Study selection flow diagram.

The PRISMA flow sheet for the search is given in Figure 1.

### 2.4. Data Extraction and Outcomes

Data extraction was conducted independently by two of the authors using a predefined abstraction form. Extracted variables included study identifiers (author names and publication year), study design, sample size, tumor types, and surgical approaches (endoscopic, open craniofacial, transcranial, or hybrid techniques) (Table 1). Additionally, data on reported outcomes (tumor resection rates, recurrence, and functional outcomes), complications (CSF leaks, infection rates, vascular injury, and morbidity), and study limitations (sample size, single-center bias, and methodological constraints) were systematically collected. Given the heterogeneity in study designs and outcome reporting, a meta-analysis was not conducted. Instead, a qualitative synthesis was performed to evaluate the comparative efficacy and feasibility of different surgical techniques in skull-based tumor management.

### 2.5. Study Design and Protocol Registration

This systematic review followed the recommendations of the Cochrane collaboration [5] and the PRISMA guidelines [6]. The study protocol was registered in the International Prospective Register of Systematic Reviews (PROSPERO) under registration number CRD42025635109. The title of this manuscript has been refined from the original PROSPERO protocol registration to better reflect the novel decision-making framework developed as a key outcome. The core research question and methodology remain unchanged from the registered protocol.

### 2.6. Ethical Considerations

No ethical issues were encountered during the study. The study was approved by the Institutional Research and Ethical Review Board (IREB) of Khyber Medical College/Khyber Teaching Hospital, Peshawar, Khyber Pakhtunkhwa, Pakistan, under approval number 548/DME/KMC, dated 08/09/2023.

## 3. Results

### 3.1. Overview of Included Studies

A total of 60 studies were included in this review, encompassing a diverse range of methodologies, sample sizes, and objectives. Study types varied widely, including retrospective analyses, prospective trials, meta-analyses, anatomical and technical reports, comparative studies, case series, and systematic reviews. Sample sizes ranged from single-patient case reports [7, 8] to large retrospective cohorts exceeding 1000 participants [9].

Among the included studies, a significant number focused on the efficacy and safety of endoscopic endonasal approaches (EEAs). These studies consistently reported CSF leak as a major complication, with rates ranging from 4% to 20% depending on surgical technique and experience level [2, 4, 10–14]. Other commonly reported complications included cranial nerve (CN) deficits—especially in transcranial approaches—ranging from 10% to as high as 60% in certain tumor resections [15–19].

Quality of life (QoL) outcomes were less frequently addressed, with only a few studies explicitly measuring patient-reported symptoms or functional outcomes postsurgery [19, 20]. Hormonal deficits and endocrine complications, including diabetes insipidus (DI), were common postoperatively in transsphenoidal pituitary surgeries, occurring in up to 20% of cases [9, 12, 21, 22]. Hyponatremia, both transient and delayed, was another concern, with rates peaking on postoperative Days 5–7 [23–25].

Several studies were notable for their comparative insights. Komotar et al. and Shahar et al., for example, highlighted lower morbidity and comparable gross total resection (GTR) rates when using endoscopic approaches compared to traditional transcranial methods [4, 26]. Likewise, Ganly et al. emphasized that high-volume centers achieved reduced complication rates, underscoring the importance of surgical experience [27].

Geographically, the literature included contributions from leading neurosurgical centers globally, reflecting evolving practices and innovations in skull base surgery. A significant body of anatomical and technical studies further complemented the clinical literature, providing foundational knowledge for safe surgical navigation of complex skull base regions [28–32].

Table 1 provides a comprehensive summary of the included studies, detailing surgical approaches, study types, sample sizes, key outcomes, complication rates, and risk of bias. It serves as the primary source of extracted data, offering an overview of the evidence base evaluated in this review.

### 3.2. Risk of Bias Assessment

The methodological quality of studies comparing endoscopic and open approaches for ASBTs was systematically evaluated using the Newcastle–Ottawa Scale (NOS), which assessed four domains: (1) selection bias (cohort representativeness and diagnostic confirmation), (2) comparability (confounder control), (3) outcome assessment (blinded evaluation), and (4) follow-up adequacy (> 80% retention). Based on these criteria, studies were categorized as low (e.g., Trooboff et al. [33] with PRISMA-compliant methods), moderate, serious (e.g., Schreckinger et al. [12] with missing data), or critical risk of bias. Notably, only Greenberg et al. [19] and Laigle-Donadey et al. [20] evaluated QoL outcomes, revealing a significant evidence gap. Two reviewers independently conducted assessments, resolving discrepancies through consensus.

Analysis revealed substantial variability in risk of bias across the literature. Low-risk studies—including systematic reviews, meta-analyses, and prospective cohorts—employed rigorous methodologies with controlled confounders (tumor size/location and surgeon experience) and standardized outcomes, providing reliable anatomical and procedural data. Moderate-risk retrospective studies offered clinically useful insights into complications (CSF leaks and CN deficits) despite lacking randomization. High-risk studies (single-center retrospectives and case reports) were compromised by selection bias, small samples, and unadjusted confounders, particularly affecting GTR and morbidity outcomes. Given that > 70% of evidence comes from retrospective designs, clinicians should prioritize low-risk studies when comparing surgical approaches. Risk of bias assessment is mentioned in Supporting File 2.

### 3.3. Comparative Outcomes of Endoscopic vs. Open Approaches by Tumor Type

To directly address the central aim of this review, we synthesized the extracted data to compare the outcomes and risk profiles of endoscopic and open approaches for the most commonly encountered ASBTs. This synthesis, presented in Table 2, moves beyond a generic comparison by stratifying the evidence based on specific tumor pathology and location. The table summarizes the preferred surgical contexts, key advantages, disadvantages, and the relative strength of evidence for each approach, providing a concise, evidence-based guide for preoperative planning.

### 3.4. QoL Comparison Between Endoscopic and Open Surgical Approaches for ASBTs

Table 3 consolidates evidence on patient-centered outcomes from the included studies. However, a critical finding of this review is the significant lack of dedicated, patient-reported QoL data in the comparative literature. Therefore, this table presents available metrics—such as recovery time, cosmetic results, and complication profiles—that indirectly influence QoL, rather than direct QoL measurements, which were rarely reported.

As summarized in Table 3, the endoscopic approach generally offers advantages in patient recovery, cosmetic outcome, and hospital stay. However, a critical finding of this review is that robust, patient-reported QoL data are severely lacking in the comparative literature. Our analysis confirms that QoL outcomes are significantly under-represented. This finding is corroborated by a recent systematic review dedicated specifically to QoL in anterior skull base surgery, which, despite its publication after our search period, underscores that this remains a critical and unresolved area requiring more standardized and prospective research [38]. The existing evidence gap makes it difficult to draw definitive conclusions about the long-term impact of these surgical approaches on patients' overall well-being.

### 3.5. The Paradigm of Midline Access: Where Endoscopy Excels

The literature delineates the EEA as a multistep procedure reliant on precise anatomical navigation [28, 29, 39]. Key technical steps, as consistently reported across studies, include preoperative imaging with CT angiography, the establishment of a binostril nasal corridor, and the creation of a nasoseptal flap for reconstruction. The approach is modular, with the specific surgical corridor (e.g., transsellar, transplanum, and transcribriform) determined by the tumor's location in the median sagittal plane, as detailed in Table 4. These modules provide access from the frontal sinus to the cervicomedullary junction, each with distinct anatomical boundaries and risk profiles.

### 3.6. The Enduring Role of Open Surgery: Managing Complexity and Lateral Extension

Complementing the endoscopic corridors, a spectrum of open approaches provides the extensive exposure necessary for managing large, lateralized, or highly vascularized ASBTs. These approaches, summarized in Table 5, form the foundational repertoire for scenarios where the endoscopic view is insufficient, underscoring the continued critical role of open techniques in the skull base surgeon's armamentarium.

### 3.7. Classification of Tumors and Epidemiology

Our analysis of the included literature identified a wide spectrum of tumors affecting the anterior skull base, each with distinct histological characteristics, epidemiological patterns, and associated complication profiles. The classification, incidence, and key surgical considerations for the most prevalent tumor types are systematically summarized in Table 6. Meningiomas and pituitary adenomas were the most frequently reported neoplasms in this region, while rarer entities such as esthesioneuroblastomas and chordomas presented unique management challenges and characteristically different risk profiles.

### 3.8. Complications

The reported complication rates for anterior skull base surgery varied considerably across the included studies, influenced by pathology, tumor size, and surgical approach. For open craniofacial resection, a large multicenter study of 1193 patients reported an overall morbidity rate of 36.3% and a mortality rate of 4.7% [27]. Predictors of complications included comorbid medical conditions, previous radiotherapy, and dural or brain invasion [27].

In contrast, analysis of endoscopic endonasal skull base surgery (ESBS) revealed a distinct complication profile. A large series of 800 procedures reported an aggregate complication rate of 19.4%, which decreased to 9.3% when CSF leaks are excluded [10]. CSF leak was the most prevalent complication, with a cumulative rate of 15.9%; however, the adoption of pedicled nasoseptal flaps reduced this rate to below 5% [1]. The major complications associated with ESBS and corresponding perioperative considerations are detailed in Table 7.

## 4. Discussion

The primary finding of this systematic review is that the choice between endoscopic and open approaches for ASBTs is not a matter of superiority of one technique over the other, but rather a nuanced decision based on a tumor's anatomical location, size, and histology, balanced against surgical goals and patient-specific factors. Our synthesis allows for the proposal of a practical framework to guide this decision.

### 4.1. Principal Findings

This systematic review synthesized evidence from 60 studies to compare endoscopic and open surgical approaches for ASBTs. The analysis reveals that the EEA is associated with favorable outcomes for midline tumors, including shorter hospital stays, faster recovery, and superior cosmetic results [1, 28, 34]. Conversely, open approaches provide critical advantages in exposure for large, lateralized, or highly vascularized tumors, facilitating radical resection at the cost of higher overall morbidity [16, 27]. Complication profiles were distinct, with EEA being characterized by a higher incidence of CSF leaks, now substantially mitigated by vascularized flaps [1, 10], while open surgery carried greater risks of significant CN deficits and soft tissue complications [16, 19].

### 4.2. Interpretation of Comparative Efficacy by Tumor Type

Our synthesis demonstrates that the optimal surgical strategy is not universal but is instead dictated by specific tumor pathology and anatomy. For pituitary adenomas and tuberculum sellae meningiomas, the EEA provides GTR rates comparable to open transcranial surgery while minimizing brain retraction and offering direct access to the sella and suprasellar cistern [4, 28]. This has established EEA as the first-line modality for these midline pathologies [2, 4]. In contrast, for large olfactory groove meningiomas or tumors with extensive invasion into the orbit or cavernous sinus, open approaches remain indispensable. The wider exposure allows for early devascularization and superior control of neurovascular structures, which is crucial for achieving GTR in these complex scenarios [16, 43]. For esthesioneuroblastomas and clival chordomas, a multimodal, often combined, approach is frequently necessary, with EEA providing excellent intranasal visualization and open approaches managing significant intracranial extension [10, 11, 44].

### 4.3. The Trade-Off: Morbidity, QoL, and Functional Outcomes

A central theme emerging from this review is the critical trade-off between the extent of resection and postoperative morbidity. While open approaches can facilitate more extensive resection for aggressive tumors, this often comes at the cost of longer recovery and higher rates of complications such as anosmia, which is nearly universal after open resection of olfactory groove meningiomas [43]. The EEA, while minimizing these issues, introduces a distinct challenge, primarily CSF leakage, though the adoption of pedicled nasoseptal flaps has reduced this rate to below 5% in many series [1, 10]. Despite the clear impact on patient experience, QoL remains significantly under-reported in the comparative literature. Our finding of this evidence gap is corroborated by a recent systematic review dedicated to QoL outcomes following skull base tumor resection, which found wide variability in methodologies and QoL instruments, limiting analyses to descriptive comparisons rather than robust, quantitative synthesis. Notably, this review revealed that the impact of surgery on QoL is often underestimated by caregivers and is more profound for patients than surgeons anticipate, with a transient decline in QoL observed across almost all studies, regardless of tumor location or surgical approach. Furthermore, the predominance of anterior skull base studies using sinonasal-specific tools such as SNOT-22 and ASBQ introduces measurement bias, as other anatomical regions and functional domains remain underexamined [38]. These findings underscore the urgent need for future prospective research employing standardized, multidimensional, disease-specific QoL instruments to fully quantify and compare the functional trade-offs between open and endoscopic approaches, thereby informing evidence-based surgical decision-making. Future studies must prioritize patient-reported outcomes to fully quantify the functional trade-offs between these surgical corridors.

### 4.4. Complication Profiles and Management Strategies

The distinct complication profiles of each approach necessitate tailored preventive strategies. In EEA, the most common complication is CSF leakage, with reported rates ranging from 4% to 20% [2, 10, 54]. The evolution of reconstruction techniques, particularly the use of vascularized pedicled flaps, has been the single most important advancement in reducing this risk [1]. ICA injury, though rare (0.2%–1%), is a dreaded complication that requires meticulous preoperative planning and emergency preparedness [53]. Open approaches, while largely avoiding postoperative CSF leaks, are associated with a higher burden of infectious complications, hematomas, and CN deficits, which can be as high as 60% in surgeries involving the cavernous sinus or petroclival region [15, 17, 19]. This underscores the importance of approach selection based on a tumor's specific risk profile.

### 4.5. A Proposed Framework for Surgical Decision-Making

**Figure 2:**
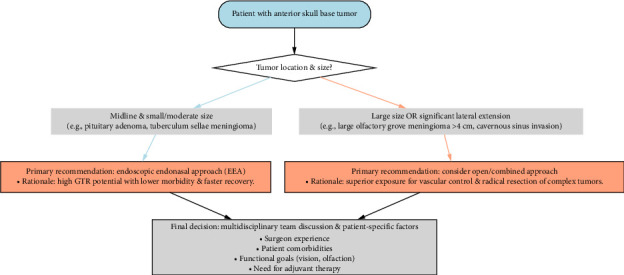
The decision-making flowchart.

Based on our comprehensive analysis of the evidence, we propose a structured decision-making framework to guide surgical approach selection for ASBTs. This framework, summarized in Figure 2, integrates tumor characteristics with surgical goals to optimize individual patient outcomes. The algorithm begins with assessment of two critical parameters: tumor location in the midline corridor and size/lateral extension, which serve as the primary determinants for initial approach selection.

As illustrated in Figure 2, the algorithm begins with an assessment of whether a tumor is primarily midline and small-to-moderate in size. If so, an endoscopic approach is recommended to leverage its benefits of reduced morbidity and comparable oncological efficacy. If the tumor is large or exhibits significant lateral extension beyond the paranasal sinuses, an open or combined approach should be strongly considered to ensure adequate exposure for safe dissection and vascular control. It is crucial to emphasize that this framework is designed not as a rigid algorithm but as a foundational guide. The final surgical plan must be determined through a multidisciplinary team discussion, incorporating critical factors such as surgeon experience, patient comorbidities and functional goals, and the need for adjunctive therapies [27, 59].

The synthesis of evidence from the included studies reveals consistent patterns in outcomes, complications, and indications for endoscopic and open approaches. To provide a clear, evidence-based overview of these comparative findings and their clinical implications, the key data are consolidated in Table 8. This summary serves as the foundational evidence for the decision-making framework proposed herein, highlighting the critical trade-offs between oncological efficacy and postoperative morbidity that must be considered during surgical planning.

## 5. Limitations

This review has some limitations. The included studies exhibited heterogeneity in design, patient populations, and outcome measures, limiting direct comparisons. Most were retrospective, single-center, and had small sample sizes, reducing generalizability. Variations in surgical techniques and inconsistent complication reporting further affected reliability. Additionally, the lack of randomized controlled trials introduced selection bias. Due to these factors, a meta-analysis was not feasible, and findings were synthesized qualitatively.

## 6. Future Directions

Future research in anterior skull base surgery should prioritize prospective, multi-institutional studies designed with standardized outcome measures. These studies must specifically integrate patient-reported QoL metrics to quantitatively assess the functional trade-offs between endoscopic and open approaches. Furthermore, high-quality comparative studies are needed to validate and refine the proposed decision-making framework, ultimately establishing more precise, evidence-based surgical indications to improve patient care in this challenging field.

## 7. Conclusion

This systematic review synthesizes 4 decades of evidence to clarify the comparative roles of endoscopic and open approaches in managing ASBTs. The analysis conclusively demonstrates that neither approach is universally superior; rather, their efficacy and safety profiles are intrinsically linked to specific tumor characteristics. The EEA has established itself as the cornerstone for midline pathologies, providing patients with the significant benefits of reduced morbidity, faster recovery, and improved QoL while maintaining high rates of GTR. Conversely, open transcranial approaches retain an indispensable role in the armamentarium, offering the extensive exposure necessary for the safe resection of large, vascular, or laterally invasive tumors.

The principal outcome of this review is a practical, evidence-based decision-making framework that empowers clinicians to navigate this complex choice. By prioritizing tumor location and size as primary selection criteria, the framework provides a clear, logical starting point for surgical planning. Ultimately, this structured approach must be integrated with a multidisciplinary team discussion, considering critical factors such as surgeon expertise, patient-specific comorbidities, and functional goals. It is through this synthesis of robust evidence and personalized clinical judgment that optimal outcomes can be achieved for patients facing these challenging diagnoses.

## Figures and Tables

**Table 1:** Summary of included studies.

Serial no.	Study (author, year)	Study design	Sample size	Surgical approach (focus)	Key findings	Complication rates	Conclusion	Risk of bias	QoL assessed
1	Patel et al. (2010)	Narrative review	N/A	Endoscopic reconstruction	Vascularized flaps superior to free grafts for reconstructive success.	CSF leak (5%–15%), infection (2%–8%)	Technique-dependent outcomes	Moderate	Yes
2	Nicolai et al. (2008)	Retrospective study	184	Endoscopic endonasal	5 year survival: 67.1% (malignant tumors)	CSF leak (6.5%), meningitis (2.2%)	Viable for sinonasal malignancies	High	No
3	Ilie et al. (2020)	Narrative review	N/A	Mixed (medical/surgical)	Aggressive adenomas: high recurrence; carcinomas: poor survival.	Hormonal deficits (30%–50%)	Multimodal therapy needed	Low	Yes
4	Komotar et al. (2012)	Comparative study	64	Endoscopic vs. transcranial	Comparable GTR rates, lower morbidity with endoscopic.	CSF leak (12.5% endoscopic)	Endoscopic reduces morbidity	Moderate	Yes
5	Cochrane Handbook (2025)	Methodology guide	N/A	N/A	PRISMA 2020 standards	N/A	N/A	N/A	N/A
6	Page et al. (2021)	Methodology Guide	N/A	N/A	PRISMA 2020 reporting standards	N/A	N/A	N/A	N/A
7	Kassam et al. (2005) (Part I)	Anatomical study	N/A (cadaveric)	Expanded endonasal	Crista galli to sella turcica feasibility demonstrated.	N/A	EEA allows ventral access	Low	No
8	Kassam et al. (2005) (Part II)	Anatomical study	N/A (cadaveric)	Expanded endonasal	Posterior clinoids to foramen magnum access demonstrated.	N/A	EEA extends to caudal skull base	Low	No
9	Prevedello et al. (2007)	Narrative review	N/A	N/A (historical)	Traced advancements in endoscopic techniques.	N/A	Endoscopy revolutionized skull base surgery	Low	No
10	Plou et al. (2023)	Anatomical study	N/A (cadaveric)	Transcranial/endonasal	Compared surgical corridors.	N/A	Anatomical insights optimize approach selection	Low	No
11	Couldwell et al. (1996)	Retrospective study	109	Transcranial	Petroclival meningiomas: 60% GTR, 20% recurrence.	CN deficits (30%), mortality (5%)	Radical resection improves outcomes	High	Yes
12	Kassam et al. (2005) (EEA clivus)	Technical report	N/A	Expanded endonasal	Middle clivus/petrous bone access described.	N/A	EEA expands ventral access	Low	No
13	Louis et al. (2016)	Classification system (WHO)	N/A	N/A	WHO CNS tumor classification published.	N/A	Standardized diagnostic criteria	Low	No
14	Al-Mefty and Borba (1997)	Retrospective study	109	Transcranial	Chordomas: 5 year survival (51%)	CN deficits (40%)	Challenging tumors requiring radical resection	High	Yes
15	Kassam et al. (2011)	Retrospective study	800	Endoscopic endonasal	Complication rate: 19.4%	CSF leak (15.9%), vascular injury (1.1%)	Safe with experience	High	Yes
16	Kassam et al. (2009)	Technical report	N/A	Expanded endonasal	Meckel's cave access feasibility.	N/A	Anteromedial corridor feasible	Low	No
17	Cavallo et al. (2005)	Anatomical study	N/A (Cadaveric)	Endoscopic endonasal	Midline skull base anatomy described.	N/A	Anatomical study guides surgery	Low	No
18	Youmans and Winn (2011)	Textbook/book chapter	N/A	N/A	Comprehensive neurosurgical techniques.	N/A	Reference for skull base approaches	Low	No
19	Laigle-Donadey et al. (2005)	Narrative review	N/A	N/A (palliative)	Skull base metastases: palliative strategies.	N/A	Focus on symptom relief	Low	Yes
20	Margalit et al. (2013)	Retrospective study	51	Transcranial/endoscopic	Tuberculum sellae meningiomas: visual improvement (80%)	CSF leak (5.9%)	Surgery improves visual outcomes	Moderate	No
21	Aguiar et al. (2009)	Retrospective study	28	Transcranial	Olfactory groove meningiomas: GTR (82%)	Anosmia (100%), CSF leak (14%)	High morbidity but effective resection	High	No
22	Lake et al. (2013)	Narrative review	N/A	N/A	Pituitary adenomas: hormonal normalization (50%–80%)	Hypopituitarism (10%–30%)	Multidisciplinary management key	Low	No
23	Müller (2014)	Narrative review	N/A	N/A	Craniopharyngiomas: 70%–90% 5 year survival	DI (80%), hypothalamic obesity (30%)	Balance resection and hypothalamic preservation	Low	No
24	Kalani et al. (2013)	Case series	12	Transcranial + revascularization	Skull base malignancies: 75% 2 year survival	Stroke (8%)	Revascularization enables radical resection	High	No
25	Sindou et al. (2007)	Retrospective study	100	Transcranial	Cavernous sinus meningiomas: 58% 10 year recurrence-free	CN deficits (38%)	Subtotal resection + radiosurgery viable	Moderate	No
26	Al-Mefty and Teixeira (2002)	Retrospective study	42	Transcranial (Fisch)	Glomus jugulare: GTR (62%)	CN deficits (45%)	Radical resection improves outcomes	High	No
27	Sivalingam et al. (2012)	Retrospective study	56	Transcranial (Fisch)	Paragangliomas: 89% control	Hearing loss (60%)	Revised Fisch classification predicts outcomes	Moderate	No
28	Almefty et al. (2007)	Retrospective study	109	Mixed	Chondrosarcomas have a better prognosis (95% 5 year survival) vs. chordomas (65%)	CN deficits (30%)	Chondrosarcomas have a better prognosis	High	No
29	Ow et al. (2013)	Narrative review	N/A	N/A	Esthesioneuroblastoma: 5 year survival (60%–80%)	Anosmia (100%)	Hyams grade predicts outcomes	Low	No
30	Ow et al. (2014)	Retrospective study	47	Craniofacial	Esthesioneuroblastoma: 10 year survival (67%)	CSF leak (13%)	Multimodal therapy improves survival	High	No
31	Diaz et al. (2005)	Retrospective study	50	Mixed	Olfactory neuroblastoma: 5 year survival (77%)	Visual loss (8%)	Kadish stage predicts survival	Moderate	No
32	Taghi et al. (2012)	Narrative review	N/A	Craniofacial	Sinonasal malignancies: 5 year survival (40%–70%)	Orbital complications (15%)	Gold standard for advanced tumors	Low	No
33	Knegt et al. (2001)	Retrospective study	22	Endoscopic + 5-FU	Ethmoid adenocarcinoma: 5 year survival (82%)	Epistaxis (9%)	Topical 5-FU reduces recurrence	High	Yes
34	Greenberg et al. (1981)	Retrospective study	43	Palliative	Skull base metastases: median survival (12 months)	CN deficits (60%)	Palliative surgery improves QoL	High	Yes
35	Ganly et al. (2005)	Multicenter study	334	Craniofacial	Major complications (36%)	CSF leak (15%), meningitis (5%)	High-volume centers reduce complications	Moderate	Yes
36	Kryzanski et al. (2002)	Narrative review	N/A	N/A	Complication avoidance strategies	Vascular injury (2%)	Preoperative planning reduces morbidity	Low	Yes
37	Kassam et al. (2011)	Retrospective study	800	Endoscopic endonasal	Complication rate: 19.4%	CSF leak (15.9%)	Safe with experience	High	No
38	Patel et al. (2010)	Narrative review	N/A	Endoscopic reconstruction	Vascularized flaps (95% success)	Flap necrosis (3%)	Vascularized flaps superior for large defects	Moderate	Yes
39	Valentine and Wormald (2011)	Narrative review	N/A	Carotid injury	Carotid injury management	Carotid injury (0.2%–1%)	Emergency preparedness critical	Low	No
40	Kim and Hong (2021)	Systematic review and meta-analysis	∼11,826 patients (pooled)	Endoscopic	CSF leak risk factors: BMI, tumor size	Pooled leak rate: 9.3%	High BMI increases leak risk	Low	No
41	Barges-Coll et al. (2010)	Anatomical study	10 cadavers	Expanded endonasal	Abducens nerve preservation landmarks identified.	CN VI palsy (0%)	Landmarks prevent nerve injury	Low	No
42	Esposito (2016)	Technical note/method description	N/A	Transsphenoidal	Standardized sella approach described.	N/A	Technique improves safety	Low	No
43	Nomikos et al. (2004)	Retrospective study	721	Transsphenoidal	Hypopituitarism improved (25%)	New hypopituitarism (15%)	Surgery can reverse deficits	High	Yes
44	Arafah (1986)	Prospective study	22	Transsphenoidal	Hormonal recovery (50%)	DI (9%)	Intrasellar pressure drives deficits	Moderate	Yes
45	Arafah et al. (2000)	Prospective study	40	Transsphenoidal	Headache relief (85%)	Transient hyponatremia (8%)	Decompression improves symptoms	Moderate	Yes
46	Kristof et al. (2009)	Prospective study	120	Transsphenoidal	DI (18%), SIADH (12%)	Electrolyte imbalances common	Close sodium monitoring needed	Moderate	Yes
47	Schreckinger et al. (2013)	Retrospective study	150	Endoscopic Transsphenoidal	Transient DI (20%)	DI correlates with tumor size	DI is often transient	High	Yes
48	Berker et al. (2012)	Retrospective study	570	Transsphenoidal	CSF leak (4%)	Lower complications with endoscopic	Endoscopic reduces morbidity	High	No
49	Cote et al. (2016)	Systematic review and meta-analysis	Pooled patients	Transsphenoidal	Delayed hyponatremia: 8.4%	Peaks at POD 5–7	Sodium monitoring critical	Low	Yes
50	Jahangiri et al. (2014)	Retrospective study	> 1000	Transsphenoidal	Endocrine improvement (40%–60%)	DI (8.2%)	Pituitary function often recovers	High	No
51	Hussain et al. (2013)	Retrospective study	200	Transsphenoidal	Delayed hyponatremia (9%)	Associated with older age	Monitor sodium post-op	High	Yes
52	Ramakrishnan et al. (2013)	Original research (observational study) (microbiome study)	28	Microbiome	Middle meatus microbiome baseline established.	N/A	Guides infection prevention strategies	Low	Yes
53	Shahangian et al. (2017)	Retrospective study	150	Endoscopic	CSF leak repair success (95%)	Reduced meningitis risk	Successful leak repair improves outcomes	Moderate	No
54	Lai et al. (2014)	Systematic review and meta-analysis	Pooled patients	Endoscopic	Meningitis rate: 0.5%	Low with antibiotics	Endoscopic surgery has low infection risk	Low	Yes
55	Brown et al. (2007)	Retrospective study	60	Endoscopic	Antibiotics reduced infection (0%)	Without antibiotics: 12% infection	Perioperative antibiotics are protective	Moderate	No
56	Spinazzi et al. (2015)	Retrospective study	120	Transsphenoidal	VTE risk post-op (3%)	Higher with longer surgery	VTE prophylaxis needed	High	No
57	Yang et al. (2022)	Retrospective study	98	Endoscopic	Higher complications in children	DI (18%), CSF leak (12%)	Pediatric patients need tailored approaches	High	No
58	Shields and Valdes-Rodriguez (1982)	Case report	1	Transsphenoidal	Tension pneumocephalus reported.	Rare but life-threatening	Early recognition critical	High	No
59	Solomiichuk et al. (2013)	Case report	1	N/A	Delayed subdural pneumocephalus reported.	N/A	Rare complication	High	No
60	Laws et al. (2016)	Methodology paper	N/A	Transsphenoidal	Proposed safety checklist	Reduces errors (e.g., vascular injury)	Checklists improve safety	Low	No

*Note:* Risk of bias: ‘Low' indicates high methodological quality; ‘High' indicates significant potential for bias (e.g., in retrospective designs, small samples, and case reports). SIADH: Syndrome of Inappropriate Antidiuretic Hormone Secretion.

Abbreviations: CN, cranial nerve; CSF, cerebrospinal fluid; DI, diabetes insipidus; EEA, endoscopic endonasal approach; GTR, gross total resection; VTE, venous thromboembolism.

**Table 2:** Comparative outcomes of endoscopic vs. open approaches by key tumor type.

Tumor type/location	Preferred approach(s) and context	Gross total resection (GTR) comparison	Key advantages	Key disadvantages/risks	Evidence level and notes
Pituitary Adenoma	Endoscopic (first-line for most, especially intrasellar and infradiaphragmatic)	Comparable to open for microadenomas; Superior/comparable for most macroadenomas.	• Superior visualization of sella and suprasellar space.	• Higher reported CSF leak rate, though significantly reduced with vascularized pedicled flaps (< 5%).	Strong/consistent. Endoscopic is now the established standard for the majority of cases.
			• Faster recovery and shorter hospital stay.	• Steep learning curve.	
			• Lower risk of nasal complications vs. open sublabial transsphenoidal.		
			• Better preserved sinonasal QoL.		

Tuberculum sellae meningioma	Endoscopic (for midline tumors with limited lateral optic canal extension).	Comparable for midline tumors.	• Superior early visual outcome due to direct decompression of the chiasm.	• Limited by significant lateral extension beyond the optic nerve.	Moderate/consistent. Optimal for tumors located in the midline; open approaches favored for complex, lateralized tumors.
			• No brain retraction.	• CSF leak risk remains a key concern (5%–20%).	
			• Avoids optic nerve manipulation.		

Olfactory groove meningioma	Open (for large tumors > 4 cm, vascular, or with significant brain invasion).	Higher GTR reported with open approaches for large tumors.	Open: Superior control for large/vascular tumors, ability to devascularize early.	Open: Anosmia (near 100%), higher overall morbidity, longer recovery.	Moderate/evolving. Choice is highly size-dependent. Endoscopic role is expanding for carefully selected smaller tumors.
	Endoscopic (for selected smaller tumors < 3 cm).		Endoscopic: Minimal invasiveness, avoids frontal lobe retraction.	Endoscopic: Challenging for tumors > 4 cm, higher risk of incomplete resection, difficult vascular control.	

Esthesioneuroblastoma	Combined (endoscopic resection + craniotomy for advanced intracranial extension).	Comparable in carefully selected stages when combined with adjuvant therapy.	Endoscopic: Excellent intranasal visualization, avoids facial incisions, en bloc possible for low stages.	Endoscopic: CSF leak risk.	Moderate/comparative. Multimodal therapy is critical. Endoscopic approaches show equivalent survival with reduced morbidity in selected cases.
	Endoscopic (For Kadish A/B, selected C).		Open: Traditional en bloc resection for extensive disease.	Open: Significant cosmetic and functional morbidities.	

Clival chordoma/chondrosarcoma	Endoscopic (primary for midline clival tumors).	Comparable to open, with trend toward higher GTR in mid-clivus.	• Direct midline access to clivus.	• Limited by lateral extension (e.g., into petrous bone, surrounding ICA).	Moderate/consistent. Endoscopic is often the first-line for midline tumors; open/combined approaches are used for extensive lateral disease.
			• No brain retraction.	• High risk of abducens nerve (CN VI) palsy.	
			• Early identification of the basilar artery.	• High CSF leak risk due to large dural defects.	

Cavernous sinus involvement	Open (for safe cranial nerve dissection and decompression) or combined approach.	Subtotal resection is common and often the goal to preserve cranial nerve function.	Open: Superior visualization and preservation of cranial nerves (III, IV, V1, and VI).	High risk of new or worsened cranial nerve deficits with either approach. Radical resection is often not the goal; surgery aims for safe decompression/debulking.	Limited/consensus. Management is often multimodal (surgery + radiosurgery). The approach is tailored to the specific compartment involved and surgical goals.
	Endoscopic (for biopsy or medial compartment disease).		Endoscopic: Access to the medial compartment.		

Craniopharyngioma	Endoscopic (for infradiaphragmatic and supradiaphragmatic midline tumors).	Comparable for prechiasmatic/intraventricular types.	Endoscopic: Direct visualization of pituitary stalk and perforating vessels, potentially reducing hypothalamic injury.	Both: High risk of panhypopituitarism and diabetes insipidus.	Moderate/comparative. Endoscopic approach is increasingly favored for midline tumors; open retains a role for highly complex cases.
	Open (for complex multilobulated or predominantly retrochiasmatic tumors).		Open: May offer better dissection planes for complex tumors adherent to hypothalamus.	Endoscopic: Challenging for tumors extending far into the third ventricle.	
				Open: Higher cognitive and neurological morbidity.	

**Table 3:** Quality of life (QoL) assessment: endoscopic vs. open approach for anterior skull base tumors.

Aspect	Endoscopic approach	Open approach	References
Patient recovery	Faster recovery due to minimally invasive nature.	Longer recovery times due to extensive surgical disruption.	Patel et al. [1], Kryzanski et al. [34], Al-Mefty and Borba [16], Ganly et al. [27], Knegt et al. [35]

Cosmetic outcome	Superior cosmetic outcomes with minimal scarring.	Visible scarring due to large incisions.	Al-Mefty and Borba [16], Patel et al. [1], Kryzanski et al. (2002) [34]

Complications	- CSF leak, meningitis risks higher.	- Higher rates of infection, hematomas.	Patel et al. [1], Ramakrishnan et al. [36], Kassam et al. [10], Knegt et al. [35], Greenberg et al. [19]
	- Risk of vascular injuries.	- Greater soft tissue complications.	

Surgical access and visualization	Better visualization in midline and skull base regions.	Better for large and laterally placed tumors.	Al-Mefty and Borba [16]

Functional outcomes	Improved functional outcomes, especially in nasal and olfactory structures.	Risk of more significant deficits, including olfactory and cranial nerve functions.	Kassam et al. [10], Laigle-Donadey et al. [20], Al-Mefty and Borba [16].

Postoperative morbidity	- Lower rates of postoperative pain and discomfort	- Higher rates of long-term morbidity due to extensive resection.	Al-Mefty and Borba [16], Barges-Coll et al. [32], Nomikos et al. [37], Laigle-Donadey et al. [20]
	- Hormonal dysfunction possible.		

Hospital stay	Shorter hospital stays and quicker return to normal activities.	Prolonged hospitalization for recovery.	Patel et al. [1], Kryzanski et al. [34], Al-Mefty and Borba [16]

Tumor recurrence	Comparable recurrence rates for smaller tumors; higher recurrence for aggressive or large tumors.	Lower recurrence for aggressive tumors due to extensive resection.	Al-Mefty and Borba [16], Komotar et al. [4], Knegt et al. [35]

Patient satisfaction	Higher satisfaction due to less visible scarring and faster recovery.	Satisfaction varies; influenced by cosmetic and functional outcomes.	Patel et al. [1], Ganly et al. [27], Knegt et al. [35]

Cost effectiveness	Generally, more cost-effective due to shorter hospital stays and fewer resources.	Higher costs due to longer hospitalization and rehabilitation.	Ganly et al. [27], Lai et al. [33], Knegt et al. [35], Al-Mefty and Borba [16]

**Table 4:** Median sagittal plane approaches in endoscopic endonasal skull base surgery [28, 29, 31, 40].

Module	Corridor	Anatomical boundary	Cistern	Neurovascular structures	Key anatomical landmark	Common pathology
Transellar	Sphenoid and posterior ethmoid	Superior intercavernous sinus to inferior intercavernous sinus, cavernous to cavernous sinus [28]	Subdiaphragmatic and suprasellar [28]	Carotid siphon; medial cavernous sinus; CNs III, IV, and VI; optic chiasm [28, 29]	Tuberculum sellae, sellar floor, “4 blues” (SIS to IIS and cavernous sinuses) [28]	RCC, pituitary adenoma [2, 4, 41]
Transplanum	Sphenoid and Posterior Ethmoid	Posterior ethmoidal artery, sella posterior, optic canals, paraclinoid ICA laterally [28]	Interhemispheric fissure [28]	Anterior circle of Willis, chiasm, optic nerves, stalk, gyrus rectus, and orbitofrontal gyrus [28]	Medial opticocarotid recess, optic canals [28, 29]	Meningioma, pituitary adenoma, craniopharyngioma [4, 26, 42]
Transcribriform	Complete ethmoid and frontal sinus “Draf III”	Back wall of frontal sinus to planum, lamina papyracea to lamina papyracea [28]	Interhemispheric fissure [28]	A2 and orbitofrontal arteries, inferior sagittal sinus, gyrus rectus, orbitofrontal gyrus [28]	Anterior and posterior ethmoidal arteries, falx, periorbita [28]	Meningioma, esthesioneuroblastoma, olfactory schwannoma [11, 43, 44]
Upper 1/3 of clivus	Sphenoid, nasopharynx, and pituitary transposition	Dorsum sellae, post clinoid, Dorello canal [29]	Anterior 3rd ventricle, interpeduncular and prepontine cistern, Liliequist membrane [29]	CN III bilat, pituitary stalk, mammillary bodies, BA, P1 and P2, PCoA, midbrain, pons [29]	Parasellar ICA, dorsum sellae, pituitary transposition, and cavernous sinus [29, 40]	Meningioma, chordoma, craniopharyngioma, pituitary adenoma [4, 16, 42]
Middle 1/3 of clivus	Sphenoid and nasopharynx	Dorello canal (sellar floor) to level of foramen lacerum [29]	Prepontine [29]	BA, posterior circle of Willis, CN VI, pons [29]	Margin, origin of abducens nerve, Dorello canal [29, 32]	Meningioma, schwannoma, chordoma, chondrosarcoma [16, 45]
Lower 1/3 of clivus	Sphenoid and nasopharynx	Foramen lacerum level through basion [29]	Prepontine, cervicomedullary [29]	CN VI bilat, CN XII bilat, VAs, medulla [29]	Vertebrobasilar junction margin, origin of abducens nerve [29]	Meningioma, chordoma, chondrosarcoma [16, 45]
Transodontoid	Nasopharynx	Basion to arch of C-1 [8]	Cervicomedullary [29]	VAs, CN XII bilat, medulla, spinal cord [29]	Eustachian tubes, odontoid ligaments, condyles [29]	Meningioma, chondrosarcoma, chordoma [16, 45]

*Note:* PCoA: posterior communicating artery.

Abbreviations: BA, basilar artery; CN, cranial nerve; ICA, internal carotid artery; IIS, inferior intercavernous sinus; RCC, Rathke's cleft cyst; SIS, superior intercavernous sinus; VA, vertebral artery.

**Table 5:** Standard open surgical approaches for anterior skull base tumor resection.

Open surgical approaches	Review
1. Craniotomy	Surgical incision on the scalp. Creation of a bone flap inside the skull. Direct access to the tumor [30].
2. Transfacial approach	Surgical incisions in the facial or neck region. Entry into the anterior skull base.
a. Transfrontal approach	Incision in the forehead. Access through frontal sinus or supraorbital area [15].
b. Transmaxillary approach	Surgical incision in the maxilla (upper jaw bone). Access through nasal cavity or maxillary sinus [40].
c. Transoral approach	Incision inside the oral cavity. Lower access to the tumor [46].
3. Trans cranial approach	Access to tumor through an incision in the skull. Removal of a section of the cranial bone.
a. Anterior craniofacial resection	Removal of the frontal bone and portions of facial bones. Access to tumor [16].
b. Orbitozygomatic approach	Excision of a segment of the zygomatic bone (cheekbone) and roof of the orbit. Access to tumor [10].
4. Endocranial approach	Use of nasal cavity as an entry point. Removal of nasal septum and associated bone.
a. Sublabial approach	Incision behind the upper lip. Access through nasal cavity [47].
b. Transsphenoidal approach	Access to tumor via sphenoid sinus (posterior to nasal cavity) [31].

**Table 6:** Classification, epidemiology, and complications of skull base tumors.

Tumor type	Description	Epidemiology and complications
Skull base tumors	Benign tumors (e.g., meningiomas, sellar/par asellar tumors, vestibular and trigeminal schwannomas) [48], Malignant tumors (e.g., chordomas, chondrosarcomas, metastases) [20].	Meningiomas: 2 cases per 100,000 individuals annually, pituitary tumors and vestibular schwannomas: 1 per 100,000 individuals [48], skull base metastases: 18 per 100,000 individuals annually [20].
1. Meningiomas	Arise from the meninges, Predominant primary tumors at the base of the skull.	5 year recurrence rates: 5% for grade I, 40% for grade II, 50%–80% for grade III.
Anterior and middle skull base meningiomas	Located in the anterior and central regions of the skull base including olfactory groove, planum sphenoidale, and tuberculum sellae meningiomas, various surgical approaches employed [26].	Anosmia (occurring in 10%–20% of cases), cerebrospinal fluid (CSF) leakage (10% incidence), and visual abnormalities and bleeding (with a prevalence of 5%–10%) [43].
2. Pituitary adenomas	Originates from the pituitary gland, classified as functional or nonfunctional adenomas [41].	Approximately 97% of microadenomas and 70% of macroadenomas have secretory activity, as shown in a study [41], surgical excision through transsphenoidal approach, possible complications: damage to internal carotid artery, optic nerve, CSF leakage.
3. Sellar and parasellar tumors	Tumors inside or close to the sella turcica region of the skull.	Transsphenoidal surgery is the preferred method.
4. Craniopharyngiomas	Benign tumors outside brain tissue but inside the arachnoid membrane, two histological subtypes: adamantinomatous (which accounts for around 95% of pediatric cases) and papillary squamous epithelium [42].	The prevalence of their occurrence is around 0.1 cases per 100,000 individuals annually. More than 80% of these tumors are in the suprasellar region [42].
5. Cavernous sinus tumors	Primary cavernous sinus meningiomas are uncommon, surgical options depend on tumor location.	Account for less than 3% of all meningiomas, current approaches involve excision for tumors outside the cavernous region or biopsy for tumors inside [17, 49].
6. Glomus tumors (paraganglioma)	Rare tumors, gradual and localized invasive growth pattern, various surgical approaches.	Have a low prevalence, occurring in around 1 to 3 instances per one million individuals and are notably more common in females, with a female-to-male ratio of 6:1 [26], surgical excision after embolization is preferred, common morbidities: facial paralysis, auditory impairment, cranial nerve palsies [50].
7. Chordomas and chondrosarcomas	Rare, benign tumors from notochordal cell lineage, limited responsiveness to radiation and chemotherapy.	The prevalence of this condition is 0.08 cases per 100,000 individuals annually, with a higher occurrence seen among men of Caucasian ethnicity [45].
8. Esthesioneuroblastoma	Uncommon neoplasm originating from olfactory epithelium, with the potential to infiltrate the cranial base, paranasal sinuses, and orbital structures.	Esthesioneuroblastomas comprise around 2%-3% of the total number of intranasal neoplasms, surgical excision is the primary treatment, complete removal improves overall outcome, common complications: CSF leakage, infection [11, 44, 51].
9. Craniofacial malignancies	Majority attributed to sinonasal malignancies, often diagnosed late, multidisciplinary approach.	Approximately 60%–70% of sinonasal malignancies originate from the maxillary sinus, while 10%–15% emerge from the ethmoid sinuses, and the remaining cases are derived from the frontal and sphenoid sinuses [52].
10. Skull base metastasis		Cranial base metastasis is observed in approximately 4% of cancer-diagnosed individuals [35]. The primary tumors most frequently associated with this condition include breast, lung, renal, and prostate cancers [19, 20].

**Table 7:** Complications and perioperative considerations in endoscopic skull base surgery.

Complications	Discussion
1. Bleeding and vascular injury	Intraoperative bleeding influenced by tumor characteristics, duration, systemic conditions, and vascularity.
	Classified as venous/arterial and low flow/high flow [53].
Low flow venous bleeding	Is often seen as widespread mucosal seeping [53].
High flow venous bleeding	Hemorrhage originating from the cavernous sinus [53].
Low flow arterial bleeding	Bleeding from tiny vessels, such as perforating vessels [53].
High flow arterial bleeding	Involves medium to large vessels, including the sphenopalatine or internal maxillary arteries, as well as the internal carotid artery (ICA) [53].
2. ICA injury	Anatomical factors that provide hazards for intraoperative damage to the internal carotid artery (ICA) include carotid dehiscence, attachment of the sphenoid septum to the ICA, and medialization of the ICA. Additional concerns include the need for revision surgery, exposure to radiation, use of bromocriptine, and acromegaly. [53].
3. CSF leaks	The most often seen postoperative complication after endoscopic skull base surgery. A comprehensive analysis of 56 research revealed that among a total of 11,826 patients who had skull base surgery, 753 individuals experienced cerebrospinal fluid (CSF) leakage. The incidence of postoperative cerebrospinal fluid (CSF) leakage was 7.2% with a 95% confidence interval ranging from 5.9% to 8.7%. The heterogeneity across the included studies was substantial, with an I2 value of 82.3%. The rate of postoperative cerebrospinal fluid (CSF) leaking remained consistent throughout different publishing years, as shown by the findings of a sensitivity study [54]. The present study conducted a thorough quantitative evaluation of postoperative cerebrospinal fluid (CSF) leakage. The findings indicated that factors such as obesity, perioperative irradiation, and high intraoperative CSF flow rate were associated with an increased likelihood of CSF leakage. Conversely, implementing a pedicled vascularized flap significantly reduced the risk of postoperative CSF leakage.
4. Neurological injury	Neural injuries in endoscopic skull base surgery (ESBS) vary from 0% to 33%, influenced by case complexity. A previous study reported an overall 1.8% neural injury rate, with 0.5% permanent cranial neuropathy, 0.8% temporary neuropathy, and 0.5% temporary hemiparesis. Permanent cases involved cranial Nerves VI, IX, X, XII, while temporary cases included cranial Nerves III, V3 motor, VI, and hemiparesis. Difficulty levels (IV and V) predicted neurological damage. Exclusive extrasellar surgery had a 2.4% neural injury rate, comparable to other procedures. Delayed deficits occurred in 1.9%, with 0.6% permanent. Collective enduring neurologic impairment was < 1% [10].
5. Abducent nerve palsy	Abducent nerve palsy, or cranial Nerve VI palsy, affects the abducens nerve, the sixth cranial nerve, leading to impaired lateral rectus muscle function and causing double vision (diplopia). This nerve is located ventrally in the cranial nerves, particularly vulnerable during transclival and paramedian surgeries. Tumor presence in the prepontine cistern can displace the nerve, increasing surgical risk. Understanding typical anatomy and anticipating abnormalities can help prevent cranial Nerve VI damage. Anatomical reference points, such as the vertebrobasilar junction, the lacerum section of the internal carotid artery (ICA), and cranial Nerve V2, aid in avoiding damage. However, these methods require precise dissection near the ICA and involve sophisticated techniques, as they pose risks to both the ICA and cranial Nerve VI's vasa nervorum [32].
6. Pituitary gland dysfunction	Diabetes insipidus (DI), syndrome of inappropriate antidiuretic hormone release (SIADH), and panhypopituitarism are potential outcomes of hypothalamic-pituitary axis (HPA) disruption due to both pathology and surgical intervention, such as the endoscopic endonasal approach (EEA) [55]. Macroadenomas are often linked to disruptions in the anterior pituitary hormone axis compared to microadenomas, attributed to portal artery compression in the infundibulum or increased intrasellar pressure [21, 23, 37]. Postoperative, HPA disturbances can result from direct or indirect manipulation [22], necessitating close clinical and laboratory monitoring to prevent complications. Transient DI occurs in 4.6%–8.7% of cases following EEA [9, 12, 13, 22, 24]. Addressing HPA dysfunction begins with preoperative assessment, adjusting cortisol levels during anesthesia induction, and considering patient characteristics associated with increased risk. During surgery, manipulating the posterior gland or infundibulum traction can heighten the risk of HPA dysregulation, particularly with pars intermedia tumors and cystic adenomas [25].
7. Infections	The presence of sinonasal microbiota and its potential connection to the cerebral cavity theoretically poses an infection risk after endoscopic endonasal surgery (EEA) [36]. However, in the absence of cerebrospinal fluid (CSF) leaks, the likelihood of meningitis or other intracranial infections is low. Failure to repair a functional CSF leak has been linked to a meningitis rate of up to 21% [14], emphasizing the importance of thorough intraoperative CSF leak repair [33]. Given the minimal infection risk and potential adverse effects of antibiotics, such as allergies, infectious colitis, and drug-resistant microbes, caution is advised in prescribing antibiotics for EEA. Studies on antibiotics in EEA primarily involve retrospective research with varying antibiotic protocols. The incidence of bacterial meningitis after EEA ranges from 0% to 0.69%, and there is no clear correlation between antibiotic selection, regimen duration, and postoperative meningitis rates. A recent trial found no significant improvement in sinonasal quality of life with postoperative prophylactic oral antibiotics [56].
7. Venous thromboembolism (VTE)	VTE is a rare occurrence in individuals undergoing endoscopic endonasal approach (EEA) procedures, but it may be more prevalent in older patients or those with coagulation issues and peripheral vascular disease. For such patients, using sequential compression devices and pharmacologic VTE prophylaxis is advisable, especially if they experience complications such as cerebrospinal fluid (CSF) leakage or cranial nerve dysfunction, which can lead to prolonged immobilization and hospital stays [57].
8. Cerebral infarction	Can result from various factors during EEA, including vasospasm, subarachnoid hemorrhage, vascular damage, or electrolyte imbalances. Some institutions employ strategies to reduce vasospasm, such as normal saline irrigation, vasodilating agents, and intravenous nimodipine postoperatively [58].
9. Pneumocephalus	The presence of air within the intracranial space can occur immediately or later after EEA. It may be due to unidirectional valves or CSF drainage. Careful lumbar drain management and sinus precautions can minimize the risk and extent of pneumocephalus [7, 8].
10. Perioperative considerations	Perioperative safety measures include nasal decongestants to reduce bleeding, image guidance for navigation, preparation of tissue graft sites, proper positioning of tubes, and optimizing surgeon ergonomics. Considerations such as inserting arterial lines or urine catheters depend on the specific procedure. Manipulating the posterior pituitary gland can lead to diabetes insipidus or syndrome of inappropriate antidiuretic hormone secretion, necessitating vigilant fluid monitoring [59].

**Table 8:** Evidence-based comparison of endoscopic vs. open approaches for anterior skull base tumors.

Aspect	Endoscopic endonasal approach (EEA)	Open transcranial approach	Clinical implications and recommendations
Optimal tumor types	• Pituitary adenomas (GTR: > 90% for microadenomas) [13, 41]	• Large olfactory groove meningiomas > 4 cm (GTR: ∼82%) [43]	First assess tumor location and size: Midline ⟶ EEA; Large/Lateral ⟶ Open. GTR is highly dependent on specific tumor anatomy.
	• Tuberculum sellae meningiomas (GTR: 80%–90%; visual improvement: > 80%) [4, 26]	• Tumors with cavernous sinus invasion (CN deficit risk: 30%–60%) [15, 17]	
	• Midline craniopharyngiomas, clival chordomas	• Tumors with significant frontal lobe involvement	

Gross total resection (GTR)	Comparable to open for midline tumors (e.g., tuberculum sellae: 80%–90% GTR) [4, 26]	Superior for large, complex tumors (e.g., olfactory groove: ∼82% GTR) [43]	GTR is achievable with both; selection depends on maximizing safety and efficacy for the specific tumor anatomy.

Common complications	• CSF leak: 4%–20% (reduced to < 5% with vascularized flaps) [1, 10, 54]	• Cranial nerve deficits: 10%–60% [15, 17, 19]	Complication profiles are distinct. EEA: Focus on robust skull base reconstruction. Open: Focus on meticulous neural preservation and dissection.
	• Vascular injury: 0.2%–1.1% [10, 53]	• Anosmia: ∼100% in olfactory groove approaches [43]	
	• Transient DI: Up to 20% [12]	• CSF leak: ∼10%–15% [27, 43]	
	• Meningitis: 0%–0.69% [33, 56]	• Infection/meningitis: Up to 5% [27]	

Recovery and morbidity	• Shorter hospital stay [1, 34]	• Longer recovery period [16, 27]	EEA offers significant short-term advantages in patient recovery and cosmetic outcomes.
	• Faster return to normal activities	• Higher overall morbidity (e.g., study reported 36% major complications) [27]	
	• Minimal visible scarring	• Visible scarring possible	
	• Lower overall morbidity rates [4]		

Quality of life impact	Superior patient-reported outcomes for cosmesis and recovery [1, 34]. However, significant evidence gap exists—only 2 of 60 included studies explicitly measured QoL [19, 20].	Variable satisfaction, often influenced by persistent functional deficits (e.g., anosmia) and cosmetic outcomes [16, 27, 43].	A major evidence gap exists. Future studies must use standardized patient-reported outcome measures [60].

Key technical and institutional factors	• Steep learning curve (complication rates drop with experience) [10]	• Familiar to most neurosurgeons	Surgeon experience, team availability, and institutional resources are critical determinants of success and should heavily influence approach selection.
	• Requires specialized equipment and a dedicated team (Neurosurgery/ENT)	• Superior for complex vascular control and wide working angles	

## Data Availability

The data supporting this systematic review are derived from publicly available studies, which are cited in the manuscript.

## Supporting Information

Supporting Information 1 Supporting File 1: Search strategies of the databases.

Supporting Information 2 Supporting File 2: Risk of bias assessment.
