# Supplementary material for: The Endoscopic Versus Open Approach for Anterior Skull Base Tumors: A Systematic Review of Comparative Outcomes and a Framework for Surgical Selection
Source: Neurol Res Int. 2025 Nov 14;2025:7730393. doi: 10.1155/nri/7730393 (PMC12638158; doi:10.1155/nri/7730393)
Supplement: Supporting Information 1 — Supporting File 1: Search strategies of the databases. [file 7730393.f1.docx]

Supplementary File 1:

| **Database** | **Search** |
| --- | --- |
| PubMed/MEDLINE | (“Anterior Skull Base Tumors”[MeSH] OR “Skull Base Neoplasms”) AND (“Endoscopic Surgery” OR “Endoscopy” OR “Minimally Invasive Surgery”) AND (“Open Surgery” OR “Craniotomy”) AND (“Complications” OR “CSF Leak” OR “Carotid Injury” OR “Meningitis”) |
| JSTOR | ("Skull Base Neoplasms" OR "Neoplasms, Skull Base" OR "Neoplasm, Skull Base" OR "Skull Base Neoplasm") AND ("Endoscopy" OR "Surgical Procedures, Endoscopic" OR "Endoscopic Surgical Procedure" OR "Endoscopic Surgical Procedures" OR "Surgical Endoscopy") AND ("Neurosurgical Procedures" OR "Neurosurgical Procedure" OR "Surgical Procedures, Neurologic" OR "Neurologic Surgical Procedure" OR "Neurologic Surgical Procedures" OR "Procedures, Neurologic Surgical") |
| Science Direct | ("Anterior Skull Base Tumors" OR "Skull Base Neoplasms") AND ("Endoscopic Approach" OR "Minimally Invasive Surgery") AND ("Open Surgery" OR "Craniotomy") AND ("CSF Leak" OR "Meningitis" OR "Infection") |
| Cochrane | ("Skull Base Neoplasms" OR "Skull Base Neoplasm" OR "Neoplasm, Skull Base")  AND ("Endoscopy" OR "Endoscopic Surgery" OR "Endoscopic Surgical Procedures" OR "Surgical Endoscopy" OR "Endoscopic Procedures") AND ("Neurosurgical Procedures" OR "Neurosurgery" OR "Neurosurgical Surgery" OR "Surgical Procedures, Neurologic" OR "Neurologic Surgery") |
| Google Scholar | “Anterior skull base tumors” AND (“Endoscopic surgery” OR “Open craniotomy”) AND (“Tumor classification” OR “Prevalence”) AND (“Surgical complications” OR “CSF leak” OR “Infection” OR “Meningitis”) |
