# Supplementary material for: The Endoscopic Versus Open Approach for Anterior Skull Base Tumors: A Systematic Review of Comparative Outcomes and a Framework for Surgical Selection
Source: Neurol Res Int. 2025 Nov 14;2025:7730393. doi: 10.1155/nri/7730393 (PMC12638158; doi:10.1155/nri/7730393)
Supplement: Supporting Information 2 — Supporting File 2: Risk of bias assessment. [file 7730393.f2.docx]

Supplementary File 2: Risk of bias assessment

| **Risk Level** | **Criteria** | **Examples from Table 1** |
| --- | --- | --- |
| **Low** | All domains met: Representative cohort, adjusted confounders, blinded outcomes, minimal attrition. | *Lai et al. (2014) [54]*: Meta-analysis with PRISMA adherence. |
| **Moderate** | 2–3 domains met: May lack adjusted confounders or blinded outcomes but otherwise robust. | *Komotar et al. (2012) [4]*: Comparative study with unmatched groups. |
| **Serious** | 1–2 domains unmet: Poor representativeness, unadjusted confounders, or high attrition. | *Schreckinger et al. (2013) [47]*: Retrospective with missing outcome data. |
| **Critical** | All domains unmet: Unconfirmed diagnoses, no controls, or >30% attrition. | *Shields & Valdes-Rodriguez (1982) [58]*: Case report with no follow-up. |
